# A New 3-Benzylchroman Derivative from Sappan Lignum (*Caesalpinia sappan*)

**DOI:** 10.3390/molecules13081923

**Published:** 2008-08-28

**Authors:** Lin-chun Fu, Xin-an Huang, Zhen-yuan Lai, Ying-jie Hu, Hong-jiao Liu, Xiao-ling Cai

**Affiliations:** 1 Tropical Medicine Institute, Guangzhou University of Chinese Medicine, Guangzhou 510405, P.R. China; E-mails: fulc01@126.com (Fu), wang2595@pchome.com.tw (Lai), yingjiehu@163.net (Hu); 2 Department of Traditional Chinese Medicine, Guangzhou Women and Children Medical Center, Guangzhou 510120, P.R. China; E-mail: hongjiao1029@163.com; 3 School of Chemistry & Chemical Engineering, Sun Yat-Sen University, Guangzhou 510275, P.R. China

**Keywords:** Sappan Lignum, chemical compounds, 3'-deoxy-4-*O*-methylepisappanol

## Abstract

3'-Deoxy-4-*O*-methylepisappanol, a new 3-benzylchroman derivative, was isolated from Sappan Lignum, together with thirteen known chemical compounds identified as protosappanin A, sappanchalcone, sappanone B, palmitic acid, (+)-(8*S*,8'*S*)-bisdihydrosiringenin, brazilein, 3-deoxysappanchalcone, (+)-lyoniresinol, 3-deoxy-sappanone B, protosappanin B, isoprotosappanin B, 3'-*O*-methylbrazilin and brazilin, respectively. Among these known compounds, this is the first time that (+)-(8*S*,8'*S*)-bis-dihydrosiringenin was obtained from the family Caesalpiniaceae.

## Introduction

**Figure 1:**
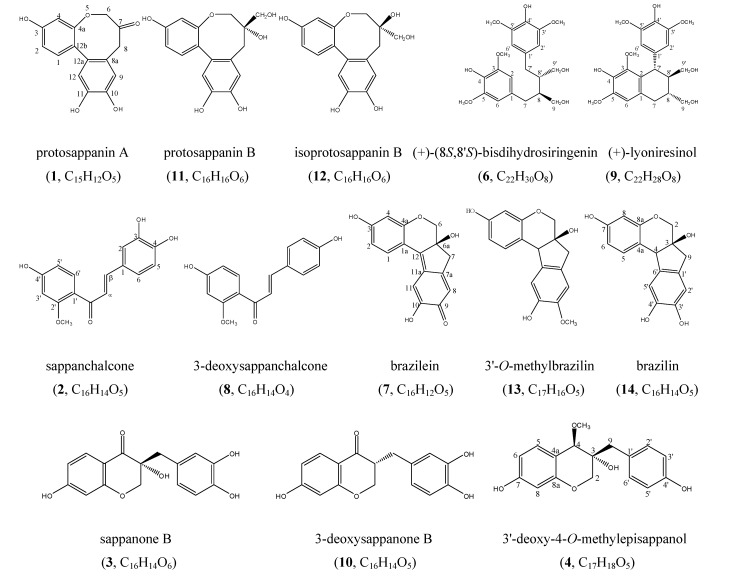
The structures of phenolic compounds from Sappan Lignum.

Sappan Lignum, the dried heartwood of *Caesalpinia sappan* L., has not only been used as a natural dyestuff for a long time [1, 2], but also a Traditional Chinese Medicine for activating blood circulation and removing stasis [3]. In recent years, the extract of Sappan Lignum has been found to be a potential immunosuppressive agent [4, 5]. The reported main phenolic compounds in Sappan Lignum were divided into to four structural sub-types: *i.e.* brazilin, chalcone, protosappanin and homisoflavonoid. Among the protosappanin derivatives, such as protosappanin B and isoprotosappanin B, 10-*O*-methy-protosappanin B and 10-*O*-methylisoprotosappanin B, as well as protosappanin E1 and protosappanin E2 occur as pairs of epimers. Meanwhile, the homisoflavonoid epimers sappanol and episappanol, 4-*O*-methylsappanol and 4-O-methylepisappanol, 3'-*O*-methylsappanol and 3'-*O*-methylepisappanol were successively isolated [6,7,8,9,10,11,12,13,14,15,16,17,18,19,20,21,22]. In continuation of our exploration of the chemical diversity of Sappan Lignum we report in this paper the isolation and identification of the new 3-benzylchroman derivative 3'-deoxy-4-*O*-methylepisappanol (**4**), and thirteen known compounds: protosappanin A (**1**), sappanchalcone (**2**), sappanone B (**3**), palmitic acid (**5**), (+)-(8*S*,8'*S*)-bisdihydrosiringenin (**6**), brazilein (**7**), 3-deoxysappanchalcone (**8**), (+)-lyoniresinol (**9**), 3-deoxysappanone B (**10**), protosappanin B (**11**), isoprotosappanin B (**12**), 3'-*O*-methylbrazilin (**13**) and brazilin (**14**) (Figure 1).

## Results and Discussion

Compound **4 **was obtained (from MeOH) as a colorless powder with the molecular formula C_17_H_18_O_5_, according to the HREIMS data. Its IR spectrum exhibited bands at 3450 (hydroxyl), 1621 and 1511 cm^-1^ (arom. ring). The ^1^H-NMR peaks at *δ*_H_ 7.16 (2H) and 6.77 (2H), and the ^13^C-NMR ones at *δ*_C_ 128.9 (s), 133.8 (d), 116.6 (d) and 158.1 (s), indicated the presence of one 1,4-disubsituted aromatic ring. A 1,2,4-trisubstituted aromatic ring was also infered from the presence of *δ*_H_ 6.96 (1H), 6.34 (1H) and 6.28 (1H) in the ^1^H-NMR spectra, and *δ*_C_ 104.5 (d), 108.9 (d), 113.6 (s), 134.3 (d), 157.2 (s) and 160.5 (s) in the ^13^C-NMR spectra (Table 1).

**Table 1:** NMR spectral data for (**4**) in acetone-*d*_6_.

No.	*δ*_H_	*δ*_C_	HMBC
2	3.82 (1H, *d*, *J =* 11.0 Hz), 4.10 (1H, *d*, *J =* 11.0 Hz)	71.2	71.1, 78.8, 157.2
3		71.7	
4	3.60 (1H, *s*)	78.8	56.9, 71.7, 134.3, 157.2
4a		113.6	
5	6.96 (1H, *d*,* J =* 8.0 Hz)	134.3	78.8, 157.2, 160.5
6	6.34 (1H, *dd*, *J =* 8.0, 2.0 Hz)	108.9	104.5, 113.6
7		160.5	
8	6.28 (1H, *d*, *J =* 2.0 Hz)	104.5	157.2, 160.5
8a		157.2	
9	2.70 (1H, *d*, *J =* 13.5 Hz), 2.91 (1H, *d*, *J =* 13.5 Hz)	40.5	71.7, 78.8, 128.9, 133.8
1′		128.9	
2′,6′	7.16 (2H, *d*,* J =* 8.5 Hz)	133.8	40.5, 133.8, 158.1
3′,5′	6.77 (2H, *d*,* J =* 8.5 Hz)	116.6	116.6, 128.9, 158.1
4′		158.1	
4-OCH_3_	3.30 (3H, *s*)	56.9	78.8

**Figure 2:**
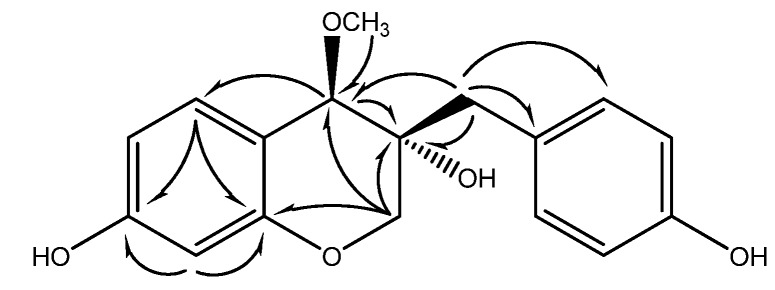
Main HMBC correlations of (**4**).

The HMBC correlations (Figure 2) between the proton at *δ*_H_ 7.16 of the 1,4-disubsituted aromatic ring and the carbon at *δ*_ C_ 40.5, the protons at *δ*_ H_ 2.70 and 2.91 to the carbon at *δ*c 78.8, the proton at *δ*_ H_ 3.60 to the carbon at *δ*_ C_ 56.9, 71.7, 134.3 and 157.2, and the protons at *δ*_ H_ 3.82 and 4.10 to the carbons at *δ*_ C_ 71.1, 78.8 and 157.2 indicated the presence of a 3,7-dihydroxy-3-(4-hydroxybenzyl)-4-methoxychroman unit. The absolute configuration at the C-3 and C-4 positions of **4** was elucidated by comparison of its ^1^H-NMR data and specific rotation with those of 4-*O*-methylsappanol and 4-*O*-methylepisappanol [13, 23]. Thus, the peaks at *δ*_H_ 3.82 and 4.10 due to the H-2 of **4** were in agreement with those of 4-*O*-methylepisappanol, while the specific rotation of **4** was – 21.0. Consequently, the configuration and structure of **4** was elucidated to be (3*R*,4*R*)-3,7-dihydroxy-3-(4-hydroxybenzyl)-4-methoxychroman, and this compounds has been named 3'-deoxy-4-*O*-methylepisappanol.

## Conclusions

3'-Deoxy-4-*O*-methylepisappanol (**4**), the epimer of 3'-deoxy-4-*O*-methylsappanol, is a new compound. (+)-(8*S*,8'*S*)-Bisdihydrosiringenin (**6**) has been observed in the family Caesalpiniaceae for the first time. Meanwhile, the epimers of protosappanin B (**11**) and isoprotosappanin B (**12**) have also also obtained from Sappan Lignum. Besides four main structural sub-types of brazilin, chalcone, protosappanin and homisoflavonoid, phenylpropanoids, including (+)-(8S,8'S)-bisdihydrosiringenin (6), (+)-lyoniresinol (9), etc., also form another structural sub-type within Sappan Lignum.

## Experimental

### General

Melting points were determined using a SGW X-4 micromelting point meter. Optical rotations were measured on a Perkin Elmer 341 polarimeter. UV absorption spectra were measured on a TU-1900 UV spectrophotometer. IR spectra were recorded on an Analect RFX-65A spectrometer. The NMR spectra were measured on a Bruker Avance 500 spectrometer (500 MHz for ^1^H and 125 MHz for ^13^C spectra), using TMS as internal standard. HREIMS and EI-MS were recorded on MAT95XP and DSQ spectrometer, respectively. Silica gel (200–300 mesh) and thin layer chromatographic plates were purchased from Qingdao Marine Chemistry Co. Ltd. (Qingdao, China). All analytical grade solvents used were obtained from Tianjin Fuyu Fine Chemical Co. Ltd. (Tianjin, China).

### Plant Material

Sappan Lignum was bought from Guangdong Medical Material Company in 2006, and authenticated by Professor Gang Hao from South China Agricultural University. The voucher specimen (Huang, 007) was deposited at Tropical Medicine Institute, Guangzhou University of Chinese Medicine.

### Extraction and Isolation

The air-dried and chipped Sappan Lignum (10 Kg) was extracted repeatedly three times under reflux with 95% EtOH. The combined EtOH extract was evaporated under reduced pressure to yield a red residue. After defatting by petroleum ether (60~90 °C), the residue was dissolved in water, then extracted successively with EtOAc and *n*-BuOH, respectively. The combined *n*-BuOH mixture (150 g) was subjected to silica gel (200~300 mesh) column, and eluted with CHCl_3_-MeOH gradient solvents. Combination of similar fractions on the basis of TLC analysis afforded fractions 1 and 2 at 97:3 and 93:7 (v/v), respectively. Fraction 1 (60 g) was chromatographed on a silica gel column eluted with petroleum ether-acetone (80:20, 70:30 and 60:40, v/v) to yield compounds **1 ** (500 mg), **2 **(1 g), **3 **(15 mg), **4 ** (3 mg), **5** (300 mg); **8** (20 mg), **10** (10 mg) and **13** (15 mg), respectively. Fraction 2 (50 g) was separated by repeated chromatography eluting with CHCl_3_-MeOH gradients of 93:7, 90:10 and 85:15 (v/v), respectively, to give compounds **6 **(15 mg), **7 **(1.5 g) and **9 **(10 mg), **11** and **12 **(100 mg).

### Compound characterization

*Protosappanin A* (**1**): was identified by comparison to literature data [24].

*Sappanchalcone* (**2**): yellow needles (from MeOH); ^13^C-NMR (acetone-*d*_6_) *δ*: 190.8 (C=O), 123.7 (C-*α*), 143.3 (C-*β*), 129.9 (C-1), 117.5 (C-2), 147.3 (C-3), 149.5 (C-4), 116.3 (C-5), 126.6 (C-6), 123.4 (C-1′), 162.7 (C-2′), 101.1 (C-3′), 164.3 (C-4′), 109.7 (C-5′), 134.4 (C-6′), 57.1 (2′-OCH_3_). Identified by the match between its ^1^H-NMR data and the reported values [23].

*Sappanone B* (**3**): colorless powder (from MeOH); ^13^C-NMR (acetone-*d*_6_) *δ*: 74.2 (C-2), 74.5 (C-3), 195.4 (C-4), 114.0 (C-4a), 131.2 (C-5), 112.9 (C-6), 166.6 (C-7), 104.6 (C-8), 165.3 (C-8a), 41.8 (C-9), 128.7 (C-1′), 116.6 (C-2′), 146.4 (C-3′), 145.9 (C-4′), 119.7 (C-5′), 124.0 (C-6′). ^1^H-NMR data was consistent with those reported [23].

*3'-Deoxy-4-O-methylepisappanol* (**4**): colorless powder (from MeOH); mp 98-99 °C; 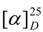 = -21.0 (*c* 0.15, MeOH), UV (MeOH) λ_max_ nm (log *ε*): 284 (3.54), 277 (3.58), 224 (4.04) nm; IR 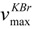
cm^-1^: 3450 (OH), 3927, 1621 and 1511 (Ar), 1382, 1162 cm^-1^; ^1^H and ^13^C-NMR spectral data were listed in Table1; EIMS *m/z* (rel. int.): [M]^+^ 302 (3), 272 (4), 153 (100), 123 (40), 107 (47), 77 (18); HREIMS *m/z*: 302.1147 [M]^+^ (calcd for C_17_H_18_O_5_, 302.1149).

*Palmitic acid* (**5**): identified by comparison to literature data [25].

*(+)-(8S,8'S)-bis-Dihydrosiringenin* (**6**): colorless powder (from MeOH); 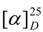 = + 10.0 (*c* 0.21, MeOH); ^1^H-NMR (pyridine-*d*_5_) *δ*: 2.51 (2H, *m*, H-8 and 8′), 3.11 (2H, *dd*, *J =* 13, 7 Hz, H-7a and 7a′), 3.15 (2H, *dd*, *J =* 13, 7 Hz, H-7b and 7b′), 3.73 (12H, *s*, 3, 3′, 5 and 5′-OCH_3_), 4.12 (2H, *dd*, *J =* 11, 5 Hz, H-9a and 9a′), 4.19 (2H, *dd*, *J =* 11, 5 Hz, H-9b and 9b′), 6.76 (4H, *s*, H-2, 2′, 6 and 6′); ^13^C-NMR (pyridine-*d*_5_) *δ*: 132.1 (C-1 and 1′), 107.5 (C-2 and 2′), 149.0 (C-3 and 3′), 135.4 (C-4 and 4′), 149.0 (C-5 and 5′), 107.5 (C-6 and 6′), 36.4 (C-7 and 7′), 44.3 (C-8 and 8′), 61.3 (C-9 and 9′), 56.3 (3, 3′, 5 and 5′-OCH_3_). The NMR spectral data were in accordance with those reported [26, 27].

*Brazilein* (**7**): identified based on its NMR data [18].

*3-Deoxysappanchalcone* (**8**): yellow needles (from MeOH); ^13^C-NMR (acetone-*d*_6_) *δ*: 190.8 (C=O), 126.6 (C-α), 142.9 (C-β), 129.2 (C-1), 131.9 (C-2 and 6), 117.7 (C-3 and 5), 161.3 (C-4), 123.4 (C-1′), 162.7 (C-2′), 101.1 (C-3′), 164.3 (C-4′), 109.7 (C-5′), 134.4 (C-6′), 57.1 (2′-OCH_3_), was confirmed asfor its ^1^H-NMR spectra, consistent with the reported data [23].

*(+)-Lyoniresinol* (**9**): colorless powder (from MeOH); 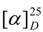 = + 2.08 (*c* 0.24, MeOH); ^1^H-NMR (pyridine-*d*_5_) *δ*: 2.24 (1H, *m*, H-8), 2.65 (1H, *m*, H-8′), 3.07 (1H, *m*, H-7a), 3.12 (1H, *m*, H-7b), 3.64 (6H, *s*, 3′ and 5′-OCH_3_), 3.76 (3H, *s*, 3-OCH_3_), 3.77 (3H, *s*, 5-OCH_3_), 4.09 (2H, *m*, H-9), 4.17 (2H, *m*, H-9′), 5.06 (1H, *d*, *J =* 5.5 Hz, H-7′), 6.78 (1H, *s*, H-6), 6.93 (1H, *s*, H-6′); ^13^C-NMR (pyridine-*d*_5_) *δ*: 129.5 (C-1), 126.6 (C-2), 147.9 (C-3), 139.4 (C-4), 148.2 (C-5), 107.4 (C-6), 33.8 (C-7), 41.6 (C-8), 66.4 (C-9), 138.4 (C-1′), 107.3 (C-2′), 148.9 (C-3′ and 5′), 135.8 (C-4′), 107.3 (C-6′), 42.3 (C-7′), 49.3 (C-8′), 64.1 (C-9′), 59.6 (3-OCH_3_), 56.0 (5-OCH_3_), 56.4 (3′ and 5′-OCH_3_) was identified based on analysis of the literature data [27,28,29].

*3-Deoxysappanone B* (**10**): colorless powder (from MeOH), 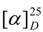 = - 10.0 (*c* 0.81, MeOH), ^1^H-NMR (acetone-*d*_6_) *δ*: 2.55 (1H, *dd*, *J =* 14.0, 10.0 Hz, H-9a), 2.81 (1H, *m*, H-3), 3.07 (1H, *dd*, *J =* 14.0, 10.0 Hz, H-9b), 4.15 (1H, *dd*, *J =* 11.0, 9.0 Hz, H-2a), 4.35 (1H, *dd*, *J =* 11.0, 9.0 Hz, H-2b), 6.37 (1H, *d*, *J =* 2.0 Hz, H-8), 6.56 (1H, *dd*, *J =* 8.0, 2.0 Hz, H-6′), 6.60 (1H, *dd*, *J =* 8.0, 2.0 Hz, H-6), 6.76 (1H, *d*, *J =* 2.0 Hz, H-2′), 6.77 (1H, *d*, *J =* 8.0 Hz, H-5′), 7.73 (1H, *d*, *J =* 8.5 Hz, H-5); ^13^C-NMR (acetone-*d*_6_) *δ*: 71.7 (C-2), 49.1 (C-3), 193.3 (C-4), 115.9 (C-4a), 130.9 (C-5), 112.3 (C-6), 166.0 (C-7), 104.4 (C-8), 165.5 (C-8a), 33.4 (C-9), 132.2 (C-1′), 117.2 (C-2′), 146.9 (C-3′), 145.5 (C-4′), 117.9 (C-5′), 122.3 (C-6′) was identified by comparison of its ^1^H-NMR data with the reported for this compound [9].

*Protosappanin B* (**11**) and *isoprotosappanin B* (**12**): colorless powders (from MeOH), were isolated as a 1.2:1 mixture; ^1^H-NMR (acetone-*d*_6_) *δ*: 2.54-2.74 (4H, each H *J* = 13 Hz, H-8), 3.16-4.35 (2H, each H *J*=11 Hz, 7-CH_2_OH), 3.16-4.35 (4H, each H*J* = 12 Hz, H-6), 6.48-6.56 (2H, *d*, *J* = 2.5 Hz, H-4), 6.56-6.62 (2H, *dd*, *J* = 8.0, 2.5 Hz, H-2), 6.71-6.84 (4H, *s*, H-9 and 12), 7.00 (2H, *d*, *J* = 8.0 Hz, H-1); ^13^C-NMR (acetone-*d*_6_) *δ*: 134.2/133.3 (C-1), 112.8/112.1 (C-2), 159.8/159.8 (C-3), 109.9/109.1 (C-4), 159.8/160.2 (C-4a), 78.2/76.9 (C-6), 73.7/73.2 (C-7), 43.6/ 40.9 (C-8), 125.9/124.3 (C-8a), 118.4 /118.3 (C-9), 145.5/145.7 (C-10), 145.5/145.5 (C-11), 119.9/120.7 (C-12), 131.1/132.6 (C-12a), 129.4/128.3 (C-12b), 66.7/69.1 ( 7-CH_2_OH). The ^1^H-NMR data ressembled those reported [23].

*3'-O-Methylbrazilin* (**13**): ^13^C-NMR (acetone-*d*_6_) *δ*: 71.8 (C-2), 78.9 (C-3), 52.4 (C-4), 116.5 (C-4a), 133.0 (C-5), 110.7 (C-6), 156.5 (C-7), 105.0 (C-8), 158.7 (C-8a), 44.0 (C-9), 132.1 (C-1′), 110.5 (C-2′), 148.7 (C-3′), 147.5 (C-4′), 113.1 (C-5′), 139.5 (C-6′), 57.5 (3′- OCH_3_). The ^1^H-NMR spectral data were identical to those reported [13].

*Brazilin* (**14**): was identified based on its NMR spectral data, identical to that reported [18].
